# Supplementary material for: Splice-Junction-Based Mapping of Alternative Isoforms in the Human Proteome
Source: Cell Rep. Author manuscript; Available in PMC 2020 Jan 15. (PMC6961840; doi:10.1016/j.celrep.2019.11.026)
Supplement: 2 [file NIHMS1546469-supplement-2.zip › DF1/PXD010154/Liver/Liver_40_RPL4_KPALCLSANESWQR.pdf]

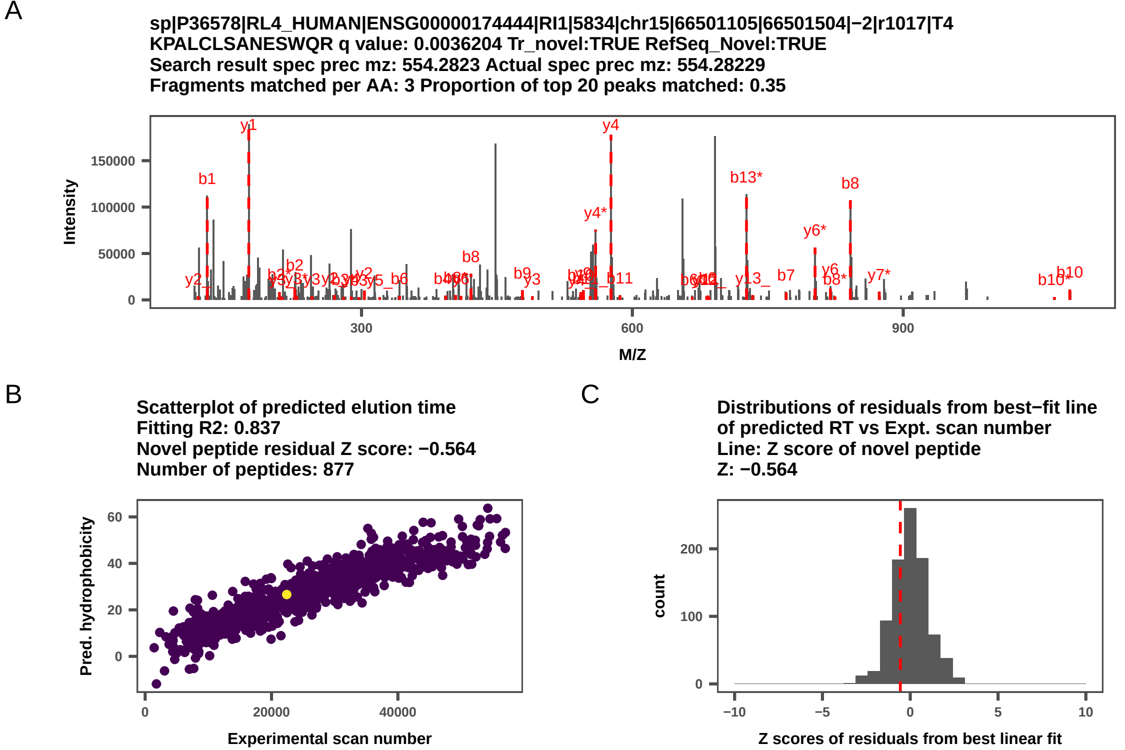

A
sp|P36578|RL4_HUMAN|ENSG00000174444|RI1|5834|chr15|66501105|66501504|−2|r1017|T4
KPALCLSANESWQR q value: 0.0036204 Tr_novel:TRUE RefSeq_Novel:TRUE
Search result spec prec mz: 554.2823 Actual spec prec mz: 554.28229
Fragments matched per AA: 3 Proportion of top 20 peaks matched: 0.35
Intensity
150000
100000
50000
0
300
600
900
M/Z
y1
b1
y4
y4*
b13*
b8
y6*
b2
b8
b9
y3
b11
y13_
b7
y6
b8*
y7*
b10
b10*
B
Scatterplot of predicted elution time
Fitting R2: 0.837
Novel peptide residual Z score: −0.564
Number of peptides: 877
Pred. hydrophobicity
60
40
20
0
20000
40000
Experimental scan number
C
Distributions of residuals from best−fit line
of predicted RT vs Expt. scan number
Line: Z score of novel peptide
Z: −0.564
count
200
100
0
−10
−5
0
5
10
Z scores of residuals from best linear fit
